# Adherence, Efficacy, and Safety of Wearable Technology–Assisted Combined Home-Based Exercise in Chinese Patients With Ankylosing Spondylitis: Randomized Pilot Controlled Clinical Trial

**DOI:** 10.2196/29703

**Published:** 2022-01-18

**Authors:** Yiwen Wang, Xingkang Liu, Weimin Wang, Yanyun Shi, Xiaojian Ji, Lidong Hu, Lei Wang, Yiquan Yin, Siyuan Xie, Jian Zhu, Jianglin Zhang, Wei Jiao, Feng Huang

**Affiliations:** 1 Department of Rheumatology and Immunology First Medical Center Chinese People's Liberation Army General Hospital Beijing China; 2 College of Sport Medicine and Sport Rehabilitation Beijing Sport University Beijing China; 3 Health Management Institute Second Medical Center Chinese People's Liberation Army General Hospital Beijing China; 4 Advanced Research Institute of Olympic Beijing Sport University Beijing China; 5 National Clinical Research Center for Orthopedics Sports Medicine and Rehabilitation Chinese People's Liberation Army General Hospital Beijing China

**Keywords:** ankylosing spondylitis, wearable technology, home-based exercise, combined exercise, randomized controlled trial, RCT, exercise, wearable, photoplethysmography, spondyloarthritis

## Abstract

**Background:**

Clinical practice guidelines recommend that exercise is essential in the self-management of ankylosing spondylitis (AS). Attending supervised interventions requiring periodic medical center visits can be difficult and patients may decline participation, whereas effective home-based exercise interventions that do not require regular medical center visits are likely to be more accessible for AS patients.

**Objective:**

The goal of the research was to investigate the adherence, efficacy, and safety of a wearable technology–assisted combined home-based exercise program in AS.

**Methods:**

This was a 16-week investigator-initiated, assessor-blinded, randomized, pilot controlled trial conducted at Chinese People’s Liberation Army General Hospital. We enrolled patients with AS who had no regular exercise habits and had been stable in drug treatment for the preceding month. Patients were randomly assigned (1:1) using a computer algorithm. An exercise program consisting of moderate-intensity aerobic exercise and functional exercise was given to the patients in the intervention group. The exercise intensity was controlled by a Mio FUSE Heart Rate Monitor wristband, which uses photoplethysmography to measure heart rate. Patients in the control group received usual care. The primary outcome was the difference in the Ankylosing Spondylitis Disease Activity Score (ASDAS). The secondary outcomes were patient global assessment (PGA), physician global assessment (PhGA), total pain, nocturnal pain, Bath Ankylosing Spondylitis Disease Activity Index (BASDAI), BAS Functional Index (BASFI), BAS Metrology Index (BASMI), Spondyloarthritis International Society Health Index (ASAS HI), 36-item Short Form Survey (SF-36), maximal oxygen uptake (VO_2_) max, body composition, range of motion of joints, and muscle endurance tests. Retention rate, adherence rate, barriers to being active, and adverse events were also assessed.

**Results:**

A total of 77 patients were screened, of whom 55 (71%) patients were enrolled; 2% (1/55) withdrew without treatment after randomization. Patients were assigned to the intervention (n=26) or control group (n=28). The median adherence rate of the prescribed exercise protocol was 84.2% (IQR 48.7%-97.9%). For the primary outcome, between-group difference of ASDAS was significant, favoring the intervention (–0.2, 95% CI –0.4 to 0.02, P=.03). For the secondary outcomes, significant between-group differences at 16 weeks were detected in PGA, PhGA, total pain, BASDAI, BASDAI-fatigue, BASDAI–spinal pain, BASDAI–morning stiffness intensity, BASFI, and BASMI. Moreover, the frequency of difficulty in ASAS HI-motivation at 16 weeks was less in the intervention group (P=.03). Between-group difference for change from baseline were also detected in VO_2_ max, SF-36, back extensor endurance test, and the range of motion of cervical lateral flexion at 16 weeks. Lack of time, energy, and willpower were the most distinct barriers to being active. Incidences of adverse events were similar between groups (P=.11).

**Conclusions:**

Our pilot study suggests that this technology-assisted combined home-based exercise program can improve the clinical outcomes of patients with AS who have no exercise habit, with good adherence and safety profile.

**Trial Registration:**

Chinese Clinical Trial Registry ChiCTR1900024244; http://www.chictr.org.cn/showproj.aspx?proj=40176

## Introduction

Ankylosing spondylitis (AS), the prototype of spondyloarthritis (SpA), is an inflammatory disease that can affect the axial skeleton and peripheral joints [1]. Since AS usually starts in young adulthood, the structural and functional impairments resulting in limitation of activities and social participation can exert considerable lifetime individual impact [2].

Clinical practice guidelines for AS recommend that exercise be included in the management of AS [3,4]. The American College of Sports Medicine (ACSM) recommends that all healthy adults participate in moderate intensity aerobic physical activity (PA) for a minimum of 30 minutes 5 days per week or vigorous intensity aerobic activity for a minimum of 20 minutes 3 days per week [5]. In 2018, a European League Against Rheumatism (EULAR) task force indicated that promoting PA consistent with general PA recommendations and conducting combined PA in the 4 domains (cardiorespiratory fitness, muscle strength, flexibility, and neuromotor performance) should be an integral part of standard care of inflammatory arthritis and osteoarthritis [6].

Although supervised exercise has advantages, it is difficult for most patients to access physiotherapists specializing in AS, and attending supervised interventions requiring periodic medical center visits can be difficult and expensive [7,8]. Conversely, home-based exercise interventions that do not require regular medical center visits are likely to be cheaper and more accessible. Currently, poor adherence and lack of monitoring strategy are 2 barriers to improving and maintaining the quality of exercise interventions [9].

Digital health encompassing a broad array of technologies has the potential to improve the quality of musculoskeletal disease care [10]. Wearable technology, one of the digital health technologies based on a wearable activity tracker (WAT), is considered to be effective to deliver exercise intervention [10]. EULAR recommendations indicate that self-monitoring tools including WAT are identified for PA assessments [6,11]. In addition, WAT can provide effective behavioral change techniques to facilitate patients to acquire skills in self-monitoring, goal setting, action planning, and feedback and problem solving in home-based exercise [6]. Currently, most evidence about WAT is for rheumatoid arthritis, osteoarthritis, and juvenile idiopathic arthritis; evidence concerning AS is scarce [10,12-15]. Therefore, we conducted this clinical trial to investigate the adherence, efficacy, and safety of this wearable technology-assisted combined home-based exercise intervention in patients with AS.

## Methods

### Study Design

This was a 16-week, randomized, open-label, accessor-blinded, controlled clinical trial conducted at the Chinese People’s Liberation Army (PLA) General Hospital (Multimedia Appendix 1). Enrolled patients were allocated with a 1:1 ratio and assessed at baseline, 8 weeks, and 16 weeks by trained research staff blinded to group assignment. The study was approved by the ethics committee at the Chinese PLA General Hospital (S2019-118-01), conducted in compliance with the Helsinki Agreement, and registered with the Chinese Clinical Trial Registry [ChiCTR 1900024244]. Informed consent was obtained from all eligible participants.

### Identification of Potential Participants

Potential participants were identified using a passive online recruitment approach through the Smartphone SpondyloArthritis Management System (SpAMS), which was created to provide patient education and deliver advice on disease management in patients with AS in China [16]. SpAMS was linked to WeChat (first released in 2011; Tencent Holdings Ltd), an instant messaging social network in China, and can be leveraged for professional purposes. Details have been published elsewhere [16,17]. As volunteers were identified, inclusion criteria were initially confirmed during online screening interviews. Written confirmation of AS diagnosis was required from the participant’s board-certified rheumatologist. If volunteers met the inclusion criteria, face-to-face interviews were conducted at the clinic for final confirmation of all inclusion criteria. Participants meeting all inclusion criteria were enrolled consecutively.

### Inclusion and Exclusion Criteria

Inclusion criteria were fulfillment of the criteria for AS (1984 Modified New York criteria) [18], aged 18 to 60 years, stable drug treatment in the preceding month, and Ankylosing Spondylitis Disease Activity Score (ASDAS) between 1.3 and 3.5. Exclusion criteria were cardiovascular disease or clinical status at high risk, screened with the American Heart Association/ACSM Health/Fitness Facility Preparticipation Screening Questionnaire [19], cervical vertebral bridges, surgery within the preceding 6 months, biological agents (tumor necrosis factor inhibitor therapy, etc) used in the preceding 3 months, regular exercise in the preceding 3 months (eg, yoga, Tai Chi, Baduanjin 3 or more times per week, 20 minutes per time), and factors leading to the inability to receive regular exercise rehabilitation (such as language impairment, difficulty in understanding, and limited movements).

### Randomization and Masking

Once the informed consent was signed and baseline assessments were completed, patients were randomly allocated to the intervention or control arm with 1:1 allocation ratio using a computer-generated randomization list performed by a research nurse not associated with the intervention portion of the study. The assessment staff and statisticians were masked to the group assignment.

### Interventions

A 16-week combined exercise program consisting of in-person counseling sessions, supervised training sessions, and aerobic and functional home-based exercise was given to patients in the intervention group after randomization.

#### In-Person Counseling Session and Brief Supervised Training Sessions

At baseline, in-person counseling sessions were held by trained research staff. The in-person counseling session is a structured interview containing the 4 domains: health benefits of exercise; overview of this exercise program; bullet points on effective and safe exercise; and how to use the wearable devices in this exercise program. In addition, supervised training sessions including a 30-minute aerobic exercise and 60 minutes of functional exercise were given for 2 consecutive days by a physiotherapist at baseline and 8 weeks to each patient assigned to the intervention group.

#### Aerobic Exercise

**Figure 1:**
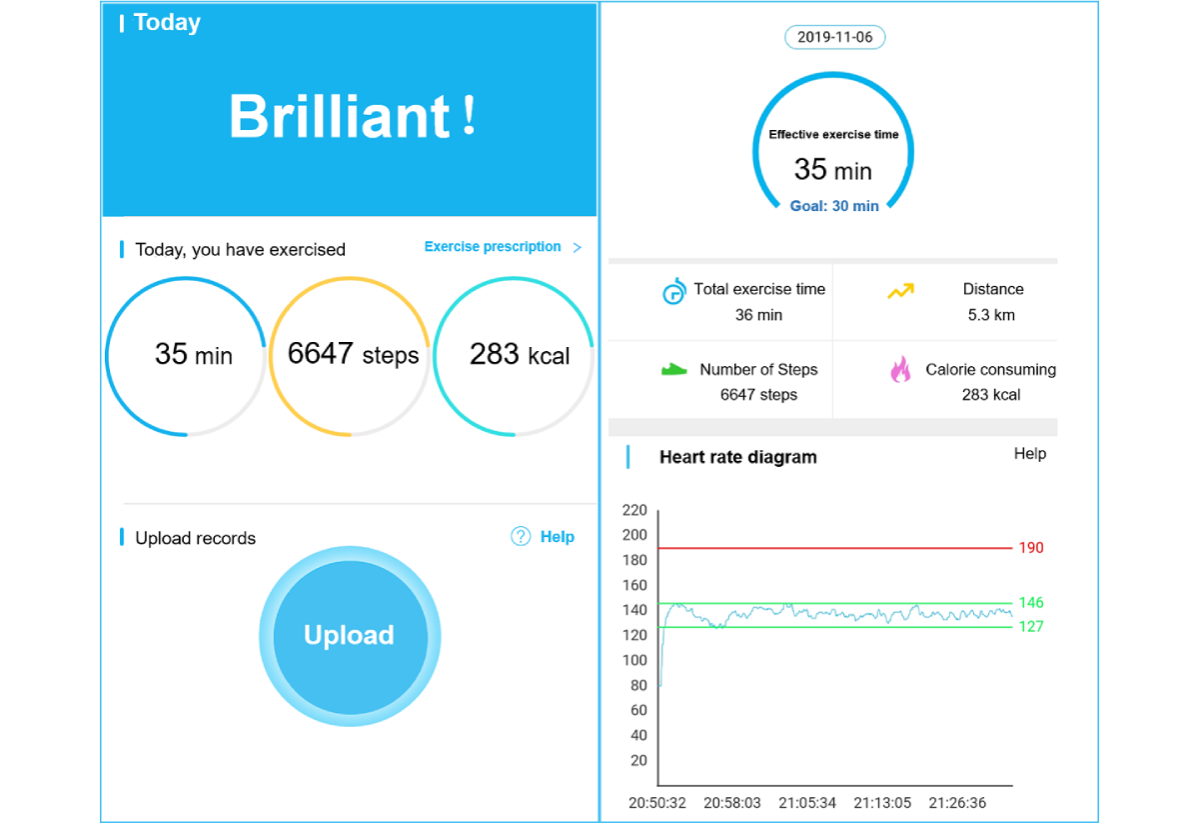
Screenshots from the treatment app. Text has been translated from Chinese to English for illustration purposes.

Aerobic exercise at a moderate intensity of 64% to 76% maximal heart rate (HRmax) was prescribed, and the exercise type was brisk walking or running (Multimedia Appendix 2) [5]. During each session, the exercise intensity was monitored and controlled by a Mio FUSE Heart Rate Monitor wristband (Medisana GmbH), which uses photoplethysmography to measure heart rate. The wristband was synchronized with the smartphone app (G health, version 2.7.1) via Bluetooth. Exercise was considered effective only when it reached moderate intensity. The prescribed protocol was 30 minutes of effective aerobic exercise 5 days per week. Data including the duration of effective aerobic exercise and heart rate during each session was all uploaded to the cloud virtual machine and the smartphone app (Figure 1).

#### Functional Exercise

Functional exercise consisting of posture, range of motion, resistance, stability, and stretching exercises was prescribed for 60 minutes 3 days per week (Multimedia Appendix 2). Individualized written functional exercise plans including key points of each movement were given to patients to help them perform home-based functional exercise and revised at the 8-week visit.

#### Usual Care

Both groups were permitted to receive routine medical therapy. Medical therapy included nonsteroidal anti-inflammatory drugs, conventional disease-modifying antirheumatic drugs including methotrexate and sulfasalazine. Biological agent or new conventional disease-modifying antirheumatic drugs were not allowed during the trial phase. Patients in the control group were asked to maintain usual PA level during the 16-week follow-up.

### Monitoring and Adherence

The sessions of aerobic exercise were recorded and analyzed based on the effective duration of exercise but not the total time spent on exercise with the consideration of better controlling the exercise intensity and duration. The duration of effective exercise was presented in the interface of the smartphone app, and the number of sessions fulfilling the exercise target was uploaded to the cloud. Adherence to the functional exercise plan was registered by uploading pictures taken after each session by the patient. At the end of the trial, the adherence rate was calculated with number of the sessions attended divided by number of sessions prescribed (128 sessions including aerobic and functional exercise sessions in 16 weeks). In addition, the Barriers to Being Active Quiz from the Centers for Disease Control and Prevention was completed by patients who participated in less than 80% of the prescribed exercise protocol in the intervention group [20].

### Outcome Assessments

An assessment was conducted at baseline (before randomization), 8 weeks, and 16 weeks by trained research staff blinded to group assignment including demographic information and primary and secondary outcomes. Investigators were blinded to outcome data until the end of the trial.

#### Primary Outcome

The primary outcome was the between-group difference for change from baseline to 16 weeks in the ASDAS [21]. ASDAS includes total back pain, patient global assessment of disease activity, peripheral pain/swelling, duration of morning stiffness, and C-reactive protein level.

#### Secondary Outcomes

In addition to the ASDAS, the Bath Ankylosing Spondylitis Disease Activity Index (BASDAI) was used to reflect disease activity. Moreover, the patient global assessment (PGA), physician global assessment (PhGA), spinal pain–total pain, spinal pain–nocturnal pain, Bath Ankylosing Spondylitis Functional Index (BASFI) [22], and Bath Ankylosing Spondylitis Metrology Index (BASMI) [23] were evaluated. Assessment using the Spondyloarthritis International Society Health Index (ASAS HI) was performed to determine the common difficulties of patients with AS [24]. Additionally, health-related quality of life was measured using the 36-Item Short Form Survey (SF-36) including physical function, role–physical, bodily pain, general health, vitality, social functioning, role–emotional, and mental health [25].

Cardiorespiratory fitness was tested with submaximal exercise tests (multistage model) on a treadmill. Based on the workload at the end of the test and estimated HRmax, the maximal oxygen uptake (VO_2_ max) was estimated [26].

Body composition including lean body mass, percentage of body fat, and visceral fat area was evaluated using the noninvasive bioelectrical impedance analysis method with the InBody 770 Body Analyzer (InBody Co, LTD).

The Clinometer smartphone app (version 3.7, Plaincode Software Solutions) was used to determine the range of motion of the cervical spine and hip joints. Back extensor and flexor endurance tests were conducted to evaluate lumbar muscle endurance.

### Sample Size

The study was designed with a planned sample size of 54 patients with a 1:1 group allocation ratio. Determination of the sample size was based on detecting a medium effect size of 0.25 calculated through a standardized mean difference of the change in ASDAS from baseline to 16 weeks between groups. Calculations were performed with G*Power (version 3.1, Heinrich Heine University) software. With a power of 95% or higher to detect differences between groups, 22 patients were calculated to be allocated to each group with a type I error rate of 5%. The loss to follow-up rate was assumed to be 20%. Therefore, the sample size of this trial was determined to be 27 patients in each group.

### Statistical Analysis

Primary and secondary outcomes were analyzed according to intention-to-treat principles by including all patients who were randomly allocated to either group and underwent at least 1 efficacy assessment. Last observation carried forward was used for missing observations. Separate analyses of covariance were used to determine mean between-group differences controlling for baseline level of outcomes. Assessments between the baseline, 8-week, and 16-week follow-up were compared using repeated-measures analysis of variance. A sensitivity analysis (per-protocol analysis) of the primary outcome was conducted with the analysis of covariance including only patients who finished the 16-week follow-up in 2 groups and who followed 80% or more of the prescribed exercise protocol in the intervention group. The chi-square or Fisher exact tests were used to compare frequencies.

Data were documented in case report forms entered into EpiData (version 4.6.0.2, EpiData Association) and analyzed using SPSS (version 24.0, IBM Corp) and GraphPad Prism 8 (GraphPad Software, Inc) software. All statistical tests were 2-sided, and P<.05 was considered to be statistically significant.

## Results

### Participants and Baseline Characteristics

**Figure 2:**
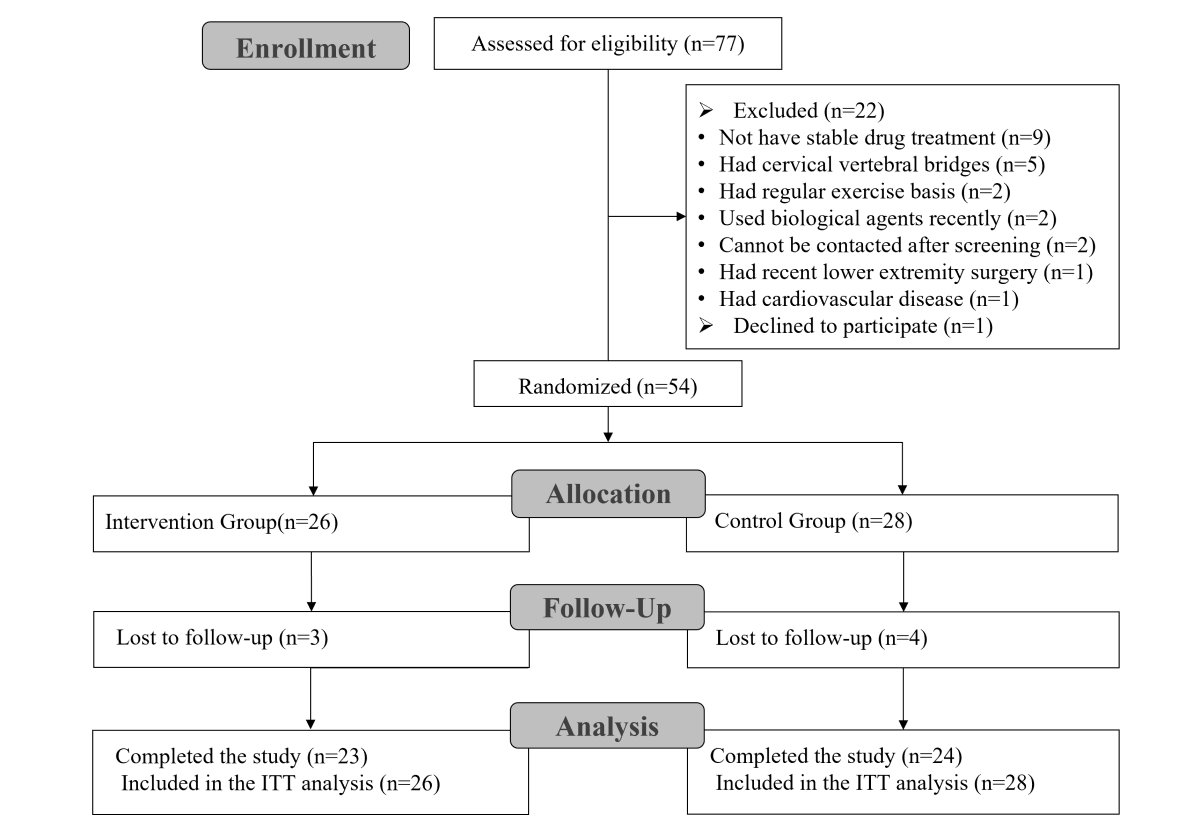
Treatment assignments and withdrawal in the intention-to-treat population. ITT: intention-to-treat.

From July to September 2019, we initially screened 77 individuals via social media, of whom 55 patients with AS were randomized in this trial. Among the patients who were randomized, one patient declined to participate due to assignment to the control group. Therefore, a total of 54 patients with AS were assigned as follows: 26 to the intervention group and 28 to the control group. The assignments and patient withdrawal data are presented in Figure 2. Table 1 shows baseline characteristics of the participants.

**Table 1:** Baseline characteristics of patients with ankylosing spondylitis enrolled in the trial.

Characteristics		Intervention group (n=26)	Control group (n=28)
Age at baseline (years), mean (SD)		31.2 (6.3)	33.2 (6.2)
Male gender, n (%)		20 (77)	21 (75)
Married, n (%)		16 (62)	18 (64)
HLA-B27^a^ positive, n (%)		24 (92)	25 (89)
Disease duration (years), mean (SD)		10.9 (5.5)	10.1 (6.1)
Height (cm), mean (SD)		173.6 (7.9)	172.0 (8.6)
Weight (kg), mean (SD)		67.7 (11.1)	70.5 (12.1)
BMI (kg/m^2^), mean (SD)		22.4 (2.8)	23.8 (3.3)
**Past history or current symptoms, n (%)**			
	AAU^b^	5 (19)	2 (7)
	IBD^c^	1 (4)	0
	Psoriasis	0	0
	Heel pain	6 (23.)	5 (18)
	Hip pain	3 (12)	4 (14)
**Physical examination, n (%)**			
	Enthesitis	6 (23)	10 (36)
	Peripheral arthritis	4 (15)	3 (11)
PGA^d^, mean (SD)		2.9 (1.5)	3.2 (1.1)
PhGA^e^, mean (SD)		2.9 (1.2)	3.2 (1.1)
Total pain, mean (SD)		2.1 (1.5)	2.8 (1.4)
Nocturnal pain, mean (SD)		2.5 (2.1)	2.5 (1.5)
ASDAS^f^, mean (SD)		1.8 (0.5)	1.7 (0.4)
BASDAI^g^, mean (SD)		2.0 (0.8)	2.2 (0.6)
BASMI^h^, mean (SD)		1.2 (1.3)	1.4 (1.7)
BASFI^i^, mean (SD)		1.1 (1.1)	1.0 (0.8)
ASAS HI^j^, mean (SD)		2.7 (2.5)	3.0 (2.9)
ESR^k^ (mm/hour)		12.8 (10.0)	10.7 (9.7)
CRP^l^ (mg/L)		5.7 (6.5)	3.0 (2.4)
NSAIDs^m^, n (%)		18 (69)	22 (79)
csDMARDs^n^, n (%)		5 (19)	8 (29)
Corticosteroids, n (%)		0	0

^a^HLA-B27: human leukocyte antigen B27.

^b^AAU: acute anterior uveitis.

^c^IBD: inflammatory bowel disease.

^d^PGA: patient global assessment.

^e^PhGA: physician global assessment.

^f^ASDAS: Ankylosing Spondylitis Disease Activity Score.

^g^BASDAI: Bath Ankylosing Spondylitis Disease Activity Index.

^h^BASMI: Bath Ankylosing Spondylitis Metrology Index.

^i^BASFI: Bath Ankylosing Spondylitis Functional Index.

^j^ASAS HI: Assessment of Spondyloarthritis International Society Health Index.

^k^ESR: erythrocyte sedimentation rate.

^l^CRP: C-reactive protein.

^m^NSAID: nonsteroidal anti-inflammatory drug.

^n^csDMARD: conventional synthetic disease-modifying antirheumatic drug.

### Retention and Adherence

The 8-week retention rates were 92% (24/26) for the intervention group and 89% (25/28) for the control group. The 16-week retention rates were 89% (23/26) for the intervention group and 86% (24/28) for the control group. The median adherence rate of the prescribed exercise protocol was 84.2% (IQR 48.7%-97.9%) among all patients assigned to the intervention group. A total of 62% (16/26) of patients in the intervention group followed 80% or more (103/128) of the prescribed exercise protocol. Only 8% (2/26) of patients attended less than 10% (13/128) of the prescribed exercise protocol.

### Primary Outcome

The between-group difference for change from baseline to 16-week follow-up of ASDAS was –0.2 (95% CI –0.4 to –0.02, P=.03), indicating more beneficial effect was detected in the intervention group (Multimedia Appendix 3).

Per-protocol analysis excluded 4 participants from the control group and 3 participants from the intervention group who were lost to follow-up and excluded 8 participants who followed less than 80% of the prescribed exercise protocol. The results were similar to primary analyses indicating a significant between-group difference in ASDAS at 16 weeks favoring the intervention (between-group difference for change from baseline, –0.3 [95% CI –0.6 to –0.1], P=.02).

### Secondary Outcome

Between-group differences for change from baseline to 16-week follow-up favoring the intervention (Multimedia Appendix 3) were observed in PGA (P<.001), PhGA (P<.001), spinal pain–total pain (P=.004), BASDAI (P=.004), BASDAI–fatigue (P=.04), BASDAI–spinal pain (P=.03), BASDAI–morning stiffness intensity (P=.04), BASDAI–morning stiffness duration (P=.05), BASFI (P=.04), and BASMI (P<.001). Moreover, the frequencies of ASAS HI–motivation (to do anything that requires physical effort) were decreased from 42% (11/26) to 19% (5/26) in the intervention group and from 50% (14/28) to 46% (13/28) in the control group at 16-week follow-up (P=.03), suggesting that this exercise program can increase motivation to do things that require physical effort (Multimedia Appendix 4). For SF-36, significant beneficial effects were detected in physical function, bodily pain, general health, and vitality in the intervention group (Multimedia Appendix 3). In addition, between-group differences for change from baseline of VO_2_ max, percentage of body fat, visceral fat area, range of motion of cervical lateral flexion and hip abduction, and back extensor endurance test were significant favoring the intervention group (Multimedia Appendix 5).

### Barriers to Being Active

**Figure 3:**
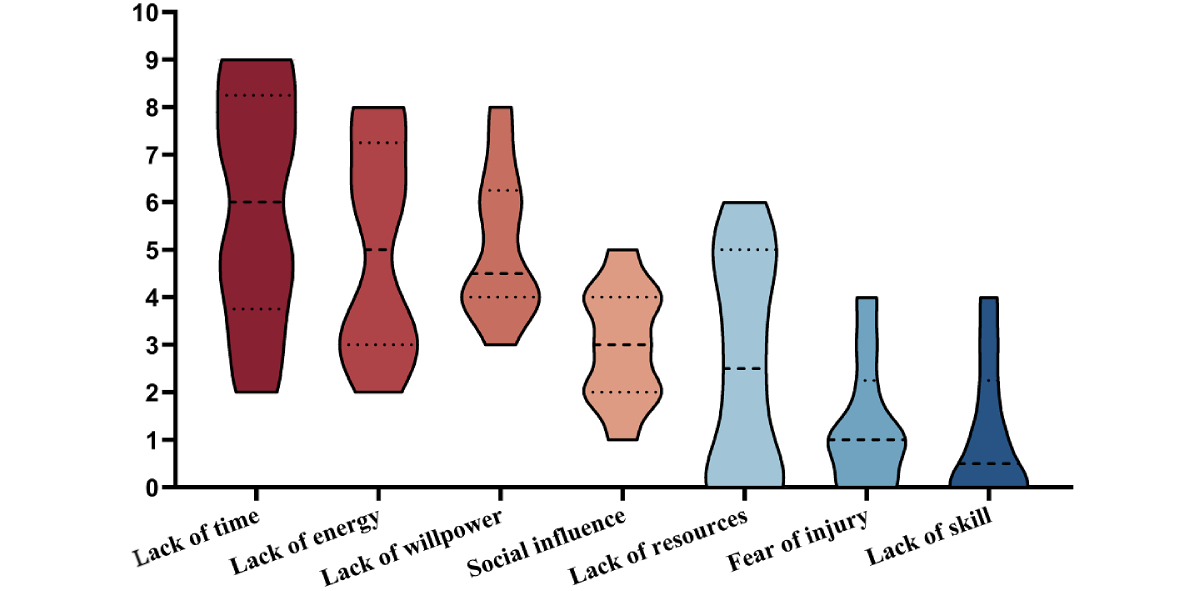
Barriers to being active. Barriers to being active were analyzed among patients who followed <80% of the prescribed exercise protocol in the intervention group.

Barriers to being active are presented in Figure 3. The results indicated that lack of time is the most distinct barrier for participants to overcome, followed by lack of energy and lack of willpower.

### Adverse Events

The incidences of adverse events observed in the intervention group and control group were 12% (3/26) and 0, respectively (P=.11), suggesting that no additional significant adverse events were caused by this exercise program. In the intervention group, 1 patient reported ankle pain and 2 patients experienced hip pain during the exercises. About 1 week after the exercise plans were adapted with less jumping movements or other adjustments, the pain disappeared, and these 3 patients all completed the intervention. No serious adverse events occurred during the trial phase in both groups.

## Discussion

### Principal Findings

In this study, we evaluated the adherence, efficacy, and safety of the wearable technology-assisted combined home-based exercise intervention in AS patients without an exercise habit through a 16-week follow-up. To our knowledge, this is the first randomized controlled trial to implement a wearable technology-assisted home-based exercise intervention in AS.

The wearable technology served as not only a supervisor but also an assessor in this exercise program, as the exercise intensity was determined and the exercise frequency and duration were assessed with the heart rate monitor embedded in the wristband. The dose of exercise reflected by type, intensity, frequency, and duration is decisive in exercise intervention [27]. In previous home-based exercise programs, PA assessments were mostly registered with the use of self-reported questionnaires or diaries, which may be influenced by patient attitudes, poor recall, and giving perceived desired responses rather than accurate ones [28]. Moreover, only class attendance can be registered, and the adherence to exercises within the attended sessions usually remains unclear [8]. In our study, the exercise type was identified, and the exercise intensity, frequency, and duration can be assessed with the heart rate monitor embedded in the wristband. For patient attitudes, a previous perspective survey of patients with axial spondyloarthritis suggested that 82.9% (97/117) of patients agreed with the implementation of exercise with heart rate monitoring, indicating the great need to explore wearable technology in the standard of care [29].

Our study proved that this wearable technology-assisted combined home-based exercise can improve the disease activity of patients with AS. In a study conducted by Hsieh et al [9] comparing the effectiveness of combined home-based exercise and range-of-motion home exercise in patients with AS, no significant improvement was observed in BASDAI in either of the 2 home-based exercise groups, but they posited this may be due to poor adherence (48%). As previous studies reported, adherence for home-based exercise was between 30% and 90%, usually in the lower range; generally, adherence with inpatient exercise was highest, followed by supervised outpatient exercise and home-based exercise [8,9]. Therefore, strategies to improve adherence of home-based exercise should be explored and identified so that the advantages of home-based exercise can be further highlighted. The good adherence rate of the prescribed exercise protocol (84%) and significant improvements of ASDAS and BASDAI in our study indicated that the use of a WAT may improve poor adherence in home-based exercise.

Identifying barriers toward applying exercise is an important issue in the research agenda of the self-management of AS [6,30]. The results of Barriers to Being Active suggested that besides lack of time, lack of energy and lack of willpower were barriers to being active. In exercise, energy and willpower can largely influence patient self-efficacy [31]. Interestingly, in our study, the improvement of BASDAI-fatigue, SF-36–VT, and ASAS HI–motivation indicated that this exercise program can help patients reduce fatigue (energy) and increase motivation to do things that requires physical effort (willpower).

In this wearable technology–assisted home-based exercise, moderate intensity exercise was prescribed considering the paradox of the benefit and harm of activity in AS. On one hand, the PA level of patients with AS should be improved, as lower PA levels are detrimental due to joint instability and loss of strength [11]. On the other hand, enthesitis is a typical feature of AS; a very high PA level is detrimental due to entheseal trauma and moving rapidly from inactivity to activity is dangerous [32]. Although high-intensity exercise is superior to moderate-intensity exercise for improving VO_2_ max [33], and previous research indicates that high-intensity exercises can improve the peak oxygen uptake (VO_2_ peak) in patients with axial spondyloarthritis over 3 months [34], the high-intensity exercise sessions should be supervised by physiotherapists throughout the trial phase. Without supervisions, it may be dangerous for AS patients considering the increased risk of injuries caused by the precipitate excessive mechanical force applied to joints [32]. Therefore, for AS patients who do not exercise on a regular basis, moderate-intensity exercise may be a more appropriate choice when performing home-based exercise.

### Limitations

Some limitations regarding the trial design merit caution. One limitation was that the patients were not blinded to the allocations, which is a common limitation of nonpharmacological treatment. To reduce bias, the primary outcome was evaluated by assessment staff who were unaware of the specific therapeutic regimen. Second, the sample size in this study was not large; however, it was determined that the sample size was sufficient as the effect size in this trial was 0.527, higher than what was set. Finally, to minimize the cofounding effect of drug treatment, only patients treated with nonsteroidal anti-inflammatory drugs and/or disease-modifying antirheumatic drugs were included in this pilot study. We have already initiated a large cohort study to determine the effect and safety of exercise in AS patients treated with different treatment strategies including biological agents.

### Conclusion

This pilot study suggests that the wearable technology–assisted combined home-based exercise is feasible and has beneficial effects on disease activity, physical function, spinal mobility, health-related quality of life, range of motion of cervical joints, and back extensor endurance in patients with AS who had no exercise habit.

## Appendix

Multimedia Appendix 1 Study protocol.

Multimedia Appendix 2 Ankylosing spondylitis exercise program used in this study.

Multimedia Appendix 3 Effects of exercise on primary outcome and other disease-related indexes.

Multimedia Appendix 4 Assessment of the Spondyloarthritis International Society Health Index was performed to indicate the common difficulties of patients with ankylosing spondylitis.

Multimedia Appendix 5 Effects of exercise on VO_2_ max, body composition, and range of motion of cervical spine and hip joints.

Multimedia Appendix 6 CONSORT-eHEALTH checklist (V 1.6.2).

## Abbreviations

ACSM: American College of Sports Medicine
AS: ankylosing spondylitis
ASAS HI: Spondyloarthritis International Society Health Index
ASDAS: Ankylosing Spondylitis Disease Activity Score
BASDAI: Bath Ankylosing Spondylitis Disease Activity Index
BASFI: Bath Ankylosing Spondylitis Functional Index
BASMI: Bath Ankylosing Spondylitis Metrology Index
DHT: digital health technology
EULAR: European League Against Rheumatism
HRmax: maximal heart rate
PA: physical activity
PGA: patient global assessment
PhGA: physician global assessment
PLA: People’s Liberation Army
SF-36: 36-item Short Form Survey
SpA: spondyloarthritis
SpAMS: Smartphone SpondyloArthritis Management System
VO_2_ max: maximal oxygen uptake
WAT: wearable activity tracker
