# Supplementary material for: Adherence, Efficacy, and Safety of Wearable Technology–Assisted Combined Home-Based Exercise in Chinese Patients With Ankylosing Spondylitis: Randomized Pilot Controlled Clinical Trial
Source: J Med Internet Res. 2022 Jan 18;24(1):e29703. doi: 10.2196/29703 (PMC8808346; doi:10.2196/29703)
Supplement: Multimedia Appendix 1 [file jmir_v24i1e29703_app1.docx]

**Protocol**

**Efficacy of Technology-assisted**

**Combined Home-based Exercise in Ankylosing Spondylitis:**

**a Randomized, Controlled trial**

**Study center**:

Department of Rheumatology, Chinese PLA General Hospital

**Principal Investigator:** Feng Huang, MD

**Contact Address**: 28 Fuxing Road, Beijing 100853, China

**Telephone**: +86-10-55499114

**Email**: fhuang@301hospital.com.cn

Table of Contents

[1 PROTOCOL SUMMARY 1](#_Toc48247241)

[1.1 Synopsis 1](#_Toc48247242)

[1.2 Schedule of Activities (SoA) 3](#_Toc48247243)

[2 INTRODUCTION 3](#_Toc48247244)

[2.1 Background 4](#_Toc48247245)

[2.2 Study Rationale 4](#_Toc48247246)

[3 Study OBJECTIVES 4](#_Toc48247247)

[3.1 Primary Endpoint 4](#_Toc48247248)

[3.2 secondary Endpoint 4](#_Toc48247249)

[4 STUDY DESIGN 6](#_Toc48247250)

[4.1 Overall Design 6](#_Toc48247251)

[5 STUDY POPULATION 6](#_Toc48247252)

[5.1 Inclusion Criteria 6](#_Toc48247253)

[5.2 Exclusion Criteria 7](#_Toc48247254)

[6 STUDY INTERVENTION 7](#_Toc48247255)

[6.1 Study Intervention Description 7](#_Toc48247256)

[6.2 Concomitant Therapy 9](#_Toc48247257)

[7 ASSESSMENT AND REPORTING OF ADVERSE EVENTS 9](#_Toc48247258)

[7.1 Adverse events (AE) 10](#_Toc48247259)

[7.2 Definition of Serious Adverse Events (SAE) 10](#_Toc48247260)

[8 STATISTICAL CONSIDERATIONS 10](#_Toc48247261)

[8.1 Sample Size Determination 11](#_Toc48247262)

[8.2 Statistical Analyses 11](#_Toc48247263)

[8.2.1 Level of statistical significance 11](#_Toc48247264)

[8.2.2 Analysis of the Primary Efficacy Endpoint(s) 12](#_Toc48247265)

[8.2.3 Analysis of the Secondary Endpoint(s) 12](#_Toc48247266)

[8.2.4 Analysis populations 12](#_Toc48247267)

[9 ETHICS 13](#_Toc48247268)

[10 REFERENCES 13](#_Toc48247269)

# PROTOCOL SUMMARY

## Synopsis

| **Title** | Efficacy of Technology-assisted combined Home-based Exercise in Ankylosing Spondylitis: a Randomized, Controlled trial |
| --- | --- |
| **Study Description** | This study was a 16-week assessor-blinded, randomized, waiting-list controlled trial (ChiCTR1900024244). Patients with ankylosing spondylitis (AS) were randomly allocated to the home-based exercise intervention group and the usual care control group. |
| **Objective** | To investigate the efficacy of a technology-assisted combined home-based exercise intervention on disease activity in patients with AS. |
| **Endpoints** | Primary Endpoint:  Ankylosing Spondylitis Disease Activity Score (ASDAS)  Secondary Endpoint:  Bath Ankylosing Spondylitis Disease Activity Index (BASDAI), Bath Ankylosing Spondylitis Functional Index (BASFI), Bath Ankylosing Spondylitis Metrology Index (BASMI), assessment of Spondyloarthritis International Society Health Index (ASAS HI), cardiorespiratory fitness, Cardiorespiratory fitness, range of motion (ROM) of cervical spine and the hip joints, cytokines |
| **Study Population** | AS patients with low or high disease activity without a regular exercise basis |
| **Description of Sites/Facilities Enrolling Participants** | A tertiary hospital in China. |
| **Description of Study Intervention** | Patients with AS were randomly allocated to the home-based exercise intervention group and the usual care group. A 16-week comprehensive exercise program consisting of a moderate intensity (64%-76% maximal heart rate [HRmax]) aerobic training for 30min on 5 days/week and a functional training for 60min on 3 days/week was given to patients in the intervention group immediately after randomization, with an in-person counseling session and supervised training sessions for two consecutive days by a study physical therapist at baseline and Week 8. The aerobic exercise intensity was controlled by a Mio FUSE Wristband with a smartphone application. Patients in control group received standard care during the 16-week follow-up and started to receive the exercise program at Week 16. |
| **Approximate Duration of Study** | 24 weeks |
| **Approximate Duration of Subject Participation** | 16 weeks |

## Schedule of Activities (SoA)

| **Procedures** | Screening  weeks -4 to 0 | Enrollment/Baseline  Visit 1, Day 1 | Study Visit 2  Week 8 +/- 5 days | Study Visit 3  Week 16 +/- 5 days |
| --- | --- | --- | --- | --- |
| Informed consent | X |  |  |  |
| Demographics | X |  |  |  |
| Clinical manifestations | X |  | X | X |
| Medical history | X |  |  |  |
| Randomization |  | X |  |  |
| Physical exam | X |  | X | X |
| Vital signs | X |  | X | X |
| ESR | X |  | X | X |
| CRP | X |  | X | X |
| BASDAI | X |  | X | X |
| BASMI | X |  | X | X |
| ASASHI | X |  | X | X |
| BASFI | X |  | X | X |
| ROM of joints |  | X | X | X |
| Cardiorespiratory fitness |  | X | X | X |
| Body composition |  | X | X | X |
| Cytokines |  | X | X | X |
| time up and go test |  | X | X | X |
| Back muscle endurance tests |  | X | X | X |
| Concomitant medication review |  |  | X | X |
| Blood routine test | X |  | X | X |
| Urine routine test | X |  | X | X |
| serum chemistry ^a^ | X |  | X | X |
| Pregnancy test ^b^ | X |  |  |  |
| Adverse event review and evaluation |  |  | X | X |
| Complete Case Report Forms (CRFs) | X | X | X | X |
| a: ALT, AST, albumin, total protein, alkaline phosphatase, γ-glutamyl-transferase, total bilirubin, direct bilirubin, glucose, BUN, creatinine.  b: Serum pregnancy test (women of childbearing potential). | | | | |

# INTRODUCTION

## Background

Ankylosing spondylitis (AS), is the prototype of spondyloarthritis (SpA), and is characterized by inflammation and new bone formation in the axial skeleton, enthesitis and peripheral arthritis^[1]^. Since AS usually starts in early adulthood, the lifetime impact of the disease can be considerable resulting in pain, stiffness, fatigue, limitation in activities and social participation^[2]^.

## Study Rationale

Clinical practice guidelines recommend that exercise is an essential component in the self-management of AS^[3]^. Attending supervised interventions requiring periodic medical center visits can be burdensome and patients may decline participation, whereas, effective home-based exercise interventions that do not need regular medical center visits are likely to be more accessible and acceptable for patients with AS. Recently, increasing evidences have been accumulated that the wearable devices could facilitate patients with inflammatory arthritis by giving exercise instructions and improving self-efficacy^[4]^. Therefore, patients with AS may benefit from an effective technology-assisted home-based exercise intervention.

# Study OBJECTIVES

## Primary Endpoint

Ankylosing Spondylitis Disease Activity Score (ASDAS) was calculated using a defined formula for evaluating the disease activity in patients with AS at baseline, 8-week and 16-week. The ASDAS_CRP_ was used as it was preferred, and when the CRP level was below the limit of detection, a constant value of 2 mg/L was used to calculate the ASDAS.

## secondary Endpoint

Disease-related clinical assessments were conducted at baseline, 8-week and 16-week. Besides ASDAS, Physician’s global assessment (PhGA), total pain, nocturnal pain, and Bath Ankylosing Spondylitis Disease Activity Index (BASDAI) was calculated to reflect patients’ disease activity. BASDAI was consisted of 6 items in fatigue, spinal pain, peripheral arthritis, enthesitis, intensity of morning stiffness, and duration of morning stiffness. Bath Ankylosing Spondylitis Functional Index (BASFI) and Bath Ankylosing Spondylitis Metrology Index (BASMI) were calculated to evaluate patients’ function status and spinal mobility, respectively. In addition, assessment of Spondyloarthritis International Society Health Index (ASAS HI) was assessed which was a health index containing 17 patient-rated items. A wide range of categories are assessed in ASAS HI including pain, emotional functions, sleep, sexual functions, mobility, self-care and community life.

Cardiorespiratory fitness was tested with submaximal exercise tests (multistage model) on treadmill at baseline, 8-week and 16-week. Three minutes rest was set between stages. Once participate reached steady-stage HRs between 115 and 150 bpm, the test was ended. Based on the workload at the end of the test and estimated HRmax, maximal oxygen uptake was estimated. Calculating the EVO2max for each workload using the ACSM metabolic equation and use the following equation to predict VO2max.

Body composition including the lean body mass, the percentage of body fat (PBF), and the visceral fat area (VFA) was evaluated with the noninvasive bioelectrical impedance analysis method by Inbody770 (Inbody Co., LTD, Seoul, Korea) at baseline, 8-week and 16-week.

The clinometer smartphone application v3.7 (Plaincodea App development) was used to determine the range of motion (ROM) of cervical spine and the hip joints at baseline, 8-week and 16-week. The time up and go test (TUGT) was used to test the patients’ functional ambulation. The back extensor and flexor endurance tests were conducted to evaluate the lumbar muscle endurance.

Peripheral blood samples were collected at baseline and 16-week and the levels of cytokines including IL-1 (α&β), IL-4, IL-6, IL-10, IL-15, IL-17 and TNF-α were evaluated at the end of the trial by Bio-Plex Pro Cytokine Assays and tested by Bio-plex 200 suspension array Luminex system (Bio-rad Laboratories, Inc.) according to the manufacturer's protocol. Specifically, at baseline, the levels of cytokines of all patients assigned to the exercise group were assessed before the first supervised training session and reassessed after finishing this session for 0.5 hours.

# STUDY DESIGN

## Overall Design

- Study Type: Interventional (Clinical Trial)
- Allocation: Randomized
- Intervention Model: Parallel Assignment
- Experimental group: Technology-assisted combined home-based exercise
- Standard group: Usual care
- Masking: Open Label
- Trial phase: 16 weeks
- Trial sites: Single site

# STUDY POPULATION

## Inclusion Criteria

- Age between 18 and 60 years;
- Fulfilling the criteria for AS (1984 Modified New York criteria );
- Drug treatment has been stable for the past 3 months;
- ASDAS score is between 1.3 and 3.5;
- Understand the purpose and procedure of this study and voluntarily sign the written informed consent;
- Patients are able to follow the schedule.

## Exclusion Criteria

- Patients with cardiovascular diseases or those at high risk;
- Patients with cervical vertebral bridges;
- Patients who underwent surgery within the last 6 months;
- Biological agents (Tumor necrosis factor inhibitors et al.) have been used in the last 3 months;
- Patients have done regular exercise in the past 3 months (e.g., yoga, Tai Chi, Baduanjin, etc.,>= 3times/weeks, 20 minutes/time);
- Patients who are unable to receive regular exercise rehabilitation due to language impairment, difficulty in understanding, limited movements and other reasons;
- Others are considered unsuitable for the study.

# STUDY INTERVENTION

## Study Intervention Description

A 16-week comprehensive exercise program consisting of in-person counseling sessions, aerobic exercise and functional exercise was given to patients in the intervention group after randomization.

***In-person counseling session and supervised training sessions***

The in-person counseling session is a structured interview containing the four domains:

- health benefits of exercise;
- overview of the exercise program;
- bullet points to effective and safe exercise;
- how to use the wearable devices in this exercise program.

In addition, supervised training sessions including a 30-min aerobic exercise and 60-min of functional exercise were given for two consecutive days by a physiotherapist at baseline and Week 8 to each patient assigned to the intervention group.

***Aerobic exercise***

Aerobic exercise at a moderate intensity of 64%-76% maximal heart rate (HRmax) was prescribed, and the exercise type was brisk walking or running^[5]^. The exercise intensity was calculated and controlled by a Mio FUSE Wristband with a smartphone application during each session. After the aerobic exercise mode was turned on, effective exercise was determined only when the heart rate was above 64% HRmax. The prescribed protocol was 30min effective aerobic exercise on 5 days/week. Data including the duration of effective aerobic exercise and heart rates during each session were all uploaded to the cloud virtual machine and the smartphone application.

***Functional exercise***

The functional exercise consisting of the posture training, range of motion exercises, strength training, stability training and stretching exercises was prescribed for 60min on 3 days/week. To make sure the efficacy and safety of the functional exercise protocol, the rating of perceived exertion (RPE) was asked after the supervised training sessions at baseline^[6]^. The functional exercise plan would be adapted if the patients’ RPE was above 14 or the pain score was above 3 (on a numerical rating scale from 0 to 10). After the supervised functional exercise, an individualized written functional exercise plan including key points of each movement was given to patients to help them perform home-based functional exercise. The functional exercise plan will be revised at the 8-week visit.

***Usual care***

Patients in control group received usual care and was asked to maintain their usual physical activity level during the 16-week follow-up. They started to receive the exercise program at Week 16.

## Concomitant Therapy

All prescription medications will be recorded on a Concomitant Medications Form including indication for use and start / stop dates. For participants in each group, biological agent or new conventional IMs were not allowed to be added during the trial phase.

# ASSESSMENT AND REPORTING OF ADVERSE EVENTS

## Adverse events (AE)

At the designated intervals for event collection, participants will be asked the following question:

“During the recent 8 weeks, were you diagnosed with any new disease or condition, or experienced significant worsening of the diseases you were previously diagnosed with?”

AEs will be obtained by self-report and will not require supporting documentation.

## Definition of Serious Adverse Events (SAE)

Serious adverse events (SAE) or Serious Adverse Drug Reaction (Serious ADR) are defined as any untoward medical occurrence that:

- is fatal,
- is life-threatening (see below),
- requires in-patient hospitalization or prolongation of existing hospitalization (see below), or
- causes persistent or significant disability / incapacity, or
- is an accidental or intentional overdose.

An SAE is considered life threatening when this places the participant at immediate risk of death from the event as it occurred. A life-threatening event does not include an event that might have caused death had it occurred in a more severe form but that did not create an immediate risk of death as it actually occurred. For example, drug-induced hepatitis that resolved without evidence of hepatic failure would not be considered life threatening, even though drug-induced hepatitis of a more severe nature can be fatal.

A hospitalization is to be considered an SAE only if it is an official admission with a duration of more than 24hr or a minimum of 2 calendar days where exact time of stay is unavailable. SAEs will be reported within 24 hours of their detection.

# STATISTICAL CONSIDERATIONS

## Sample Size Determination

The study was designed with a planned sample size of 54 patients with a 1:1 group allocation ratio. Sample size for the primary outcome, change in ASDAS from baseline to 16 weeks after randomization, was based on detecting a medium effect size of 0.25 and was performed with the G*Power 3.1 software. With a power of 95% or higher to detect differences between groups, 22 patients were calculated to be allocated to each group with a type I error rate of 5%. The loss to follow-up rate was assumed to be 20%; therefore, the sample size of this trial was determined to be 27 patients in each group.

## Statistical Analyses

### Level of statistical significance

The primary and secondary outcomes were analyzed according to the intention-to-treat (ITT) principles by including all patients who were randomly allocated to either group and underwent at least 1 efficacy assessment, without considering their actual compliance with intervention. Last observation carried forward was used for missing observations. The mean difference between groups in change from baseline was analyzed with the analysis of covariance. Separate analyses of covariance were used to compare mean change from baseline and to determine mean between group differences controlling for baseline level of outcome. A sensitivity analysis (per-protocol analysis) of primary outcome was conducted including only patients who finished the 16-week follow-up and those in the intervention group who followed ≥80% of the prescribed exercise protocol. The χ2 or Fisher exact tests were used to compare frequencies. The levels of cytokines before and after the first supervised training session of exercise were analyzed with paired t test or Wilcoxon signed-rank test.

Data was documented in case report forms, entered into Epidata 4.6.0.2 and analyzed using SPSS version 24.0 (IBM, Armonk, North Castle, NY, USA) and GraphPad Prism 8 (GraphPad Software, Inc., La Jolla, CA, USA) software. All statistical tests were 2-sided with a significance level of α set at .05.

### Analysis of the Primary Efficacy Endpoint(s)

The primary outcome was analyzed according to the ITT principles by including all patients who were randomly allocated to either group and underwent at least 1 efficacy assessment, without considering their actual compliance with intervention. Last observation carried forward was used for missing observations. The mean difference between groups in change from baseline was analyzed with the analysis of covariance. Separate analyses of covariance were used to compare mean change from baseline and to determine mean between group differences controlling for baseline ASDAS. A sensitivity analysis (per-protocol analysis) of primary outcome was conducted including only patients who finished the 16-week follow-up and those in the intervention group who followed ≥80% of the prescribed exercise protocol.

### Analysis of the Secondary Endpoint(s)

The secondary outcomes were analyzed according to ITT with last observation carried forward. The mean difference between groups in change from baseline was analyzed with the analysis of covariance. Separate analyses of covariance were used to compare mean change from baseline and to determine mean between group differences controlling for baseline level of outcome. The χ2 or Fisher exact tests were used to compare frequencies. The levels of cytokines before and after the first supervised training session of exercise were analyzed with paired t test or Wilcoxon signed-rank test.

### Analysis populations

Analyses will be performed on the intention-to-treat population including all patients who will be randomly allocated to either treatment group and underwent at least one efficacy assessment, without regard to their actual compliance with treatment.

# ETHICS

The study protocol will be conducted in accordance with the ethical principles. Informed consent will be obtained from all the enrolled patients.

# REFERENCES

[1] Taurog JD, Chhabra A, Colbert RA. Ankylosing Spondylitis and Axial Spondyloarthritis[J]. N Engl J Med, 2016, 374(26): 2563-2574.

[2] Millner JR, Barron JS, Beinke KM, et al. Exercise for ankylosing spondylitis: An evidence-based consensus statement[J]. Semin Arthritis Rheum, 2016, 45(4): 411-427.

[3] van der Heijde D, Ramiro S, Landewe R, et al. 2016 update of the ASAS-EULAR management recommendations for axial spondyloarthritis[J]. Ann Rheum Dis, 2017, 76(6): 978-991.

[4] Davergne T, Pallot A, Dechartres A, et al. Use of Wearable Activity Trackers to Improve Physical Activity Behavior in Patients With Rheumatic and Musculoskeletal Diseases: A Systematic Review and Meta-Analysis[J]. Arthritis Care Res (Hoboken), 2019, 71(6): 758-767.

[5] Garber CE, Blissmer B, Deschenes MR, et al. American College of Sports Medicine position stand. Quantity and quality of exercise for developing and maintaining cardiorespiratory, musculoskeletal, and neuromotor fitness in apparently healthy adults: guidance for prescribing exercise[J]. Med Sci Sports Exerc, 2011, 43(7): 1334-1359.

[6] Williams N. The Borg Rating of Perceived Exertion (RPE) scale[J]. Occupational Medicine, 2017, 67(5): 404-405.
