# Supplementary material for: Adherence, Efficacy, and Safety of Wearable Technology–Assisted Combined Home-Based Exercise in Chinese Patients With Ankylosing Spondylitis: Randomized Pilot Controlled Clinical Trial
Source: J Med Internet Res. 2022 Jan 18;24(1):e29703. doi: 10.2196/29703 (PMC8808346; doi:10.2196/29703)
Supplement: Multimedia Appendix 2 [file jmir_v24i1e29703_app2.doc]

|  | |
| --- | --- |
|  | |
| **Table S1**  **AS exercise program (Intervention)**  Exercise time: 16 weeks | |
| **Aerobic exercise** | |
| Type  Time  Intensity  Frequency  Progression  Supervision method | Brisk walking or running  30 min  moderate intensity (64%-76% HR max)  5 times per week  Based on Cardiovascular test at 8 week, the target heart rate zone was adapted.  The intensity was controlled by a Mio FUSE Wristband with a smartphone application during each session. |
| **Functional exercise** | |
| Type  Pattern  Intensity  Sets  Time  Frequency  Progression  Supervision method | Five kinds of personalized functional exercise that includes posture training, range of motion exercise, strength training, stability training and stretching exercise.  Circle of exercise, 30s rest between each exercise.  8-10 repetitions of overcoming body weight  2 sets  60 min  3 times per week  If the patient could perform the exercise without any effort (Rate of perceived exertion scale was less than 9), the exercise program was adapted, workload was increased.  WeChat |
