# Supplementary material for: Adherence, Efficacy, and Safety of Wearable Technology–Assisted Combined Home-Based Exercise in Chinese Patients With Ankylosing Spondylitis: Randomized Pilot Controlled Clinical Trial
Source: J Med Internet Res. 2022 Jan 18;24(1):e29703. doi: 10.2196/29703 (PMC8808346; doi:10.2196/29703)
Supplement: Multimedia Appendix 3 [file jmir_v24i1e29703_app3.docx]

Multimedia Appendix 3. Effects of exercise on primary outcome and other disease-related indexes.

| **Outcome** | **Visit** | **Intervention Group, n=26** | | **Control Group, n=28** | | **Mean Between-Group Difference for Change from Baseline b** | ***P* Value b** |
| --- | --- | --- | --- | --- | --- | --- | --- |
|  |  | **Mean Change from Baseline** | ***P* Valuea** | **Mean Change from Baseline** | ***P* Valuea** |  |  |
| ASDAS | 8wk | -0.3 (-0.6, -0.1) | **.001** | 0.04 (-0.2, 0.3) | >.99 | -0.3 (-0.5, -0.1) | **.01** |
|  | 16wk | -0.4 (-0.6, -0.2) | **<.001** | -0.1 (-0.3, 0.1) | .73 | -0.2 (-0.4, -0.02) | **.03** |
| PGA | 8wk | -1.4 (-1.8, -1.0) | **<.001** | -0.3 (-0.7, -0.1) | .46 | -1.1 (-1.7, -0.5) | **<.001** |
|  | 16wk | -1.6 (-1.9, -1.3) | **<.001** | -0.6 (-1.0, -0.3) | **.02** | -0.9 (-1.4, -0.5) | **<.001** |
| PhGA | 8wk | -1.3 (-1.9, -0.7) | **<.001** | -0.5 (-1.0, 0.1) | .14 | -1.0 (-1.5, -0.6) | **<.001** |
|  | 16wk | -1.5 (-2.1, -0.9) | **<.001** | -0.8 (-1.3, -0.2) | **.009** | -1.0 (-1.4, -0.5) | **<.001** |
| Total pain | 8wk | -0.9 (-1.6, -0.1) | **.01** | -0.8 (-0.5, -0.1) | **.03** | -0.4 (-1.1, 0.3) | .22 |
|  | 16wk | -1.3 (-2.0, -0.5) | **<.001** | -0.9 (-1.6, -0.1) | **.01** | -0.8 (-1.3, -0.3) | **.004** |
| Nocturnal pain | 8wk | -1.6 (-2.6, -0.7) | **<.001** | -0.9 (-1.8, 0.1) | .07 | -0.7 (-1.5, -0.03) | .06 |
|  | 16wk | -1.4 (-2.3, -0.5) | **0.001** | -1.1 (-1.0, -0.3) | **.005** | -0.2 (-0.9, 0.4) | .41 |
| BASDAI | 8wk | -0.9 (-1.3, -0.5) | **<0.001** | -0.4 (-0.8, -0.04) | **.02** | -0.6 (-1.0, -0.2) | **.002** |
|  | 16wk | -1.1 (-1.5, -0.7) | **<0.001** | -0.7 (-1.1, -0.3) | **<.001** | -0.5 (-0.9, -0.2) | **.004** |
| BASDAI  -fatigue | 8wk | -0.7 (-1.6, 0.2) | .15 | -0.7 (-0.6, 0.1) | .11 | -0.6 (-1.5, 0.2) | .14 |
|  | 16wk | -1.2 (-2.1, -0.2) | **.02** | -0.9 (-1.8, 0.1) | .09 | -1.0 (-2.0, -0.1) | **.04** |
| BASDAI  -spinal pain | 8wk | -1.5 (-2.2, -0.8) | **<.001** | -0.6 (-1.3, 0.04) | .07 | -0.7 (-1.4, -0.04) | **.04** |
|  | 16wk | -1.5 (-2.2, -0.9) | **<.001** | -0.8 (-1.4, -0.1) | **.02** | -0.7 (-1.3, -0.1) | **.03** |
| BASDAI  -peripheral arthritis | 8wk | -0.4 (-1.1, 0.3) | .52 | 0.1 (-0.6, 0.8) | >.99 | -0.5 (-1.0, 0.1) | .12 |
|  | 16wk | -0.5 (-1.0, 0.04) | .08 | -0.2 (-0.7, 0.3) | >.99 | -0.3 (-0.7, 0.1) | .14 |
| BASDAI  -enthesitis | 8wk | -0.9 (-1.7, -0.1) | **.03** | -0.4 (-0.2, 0.4) | .83 | -0.4 (-1.1, 0.3) | .24 |
|  | 16wk | -1.2 (-2.1, -0.3) | **.008** | -0.8 (-1.6, 0.1) | .11 | -0.2 (-0.8, 0.3) | .39 |
| BASDAI  -morning stiffness intensity | 8wk | -1.2 (-1.7, -0.7) | **<.001** | -0.6 (-1.1, 0.0) | **.03** | -0.8 (-1.3, -0.3) | **.002** |
|  | 16wk | -1.2 (-1.8, -0.5) | **<.001** | -1.0 (-1.6, -0.4) | **<.001** | -0.5 (-0.9, -0.03) | **.04** |
| BASDAI  -morning stiffness duration | 8wk | -1.0 (-1.7, -0.3) | **.002** | -0.4 (-1.1, 0.3) | .46 | -0.8 (-1.5, -0.1) | **.02** |
|  | 16wk | -1.0 (-1.6, -0.3) | **.001** | -0.7 (-1.3, -0.1) | **.02** | -0.5 (-1.0, 0.01) | .054 |
| BASFI | 8wk | -0.4 (-0.8, -0.02) | **.03** | -0.2 (-0.6, 0.1) | .39 | -0.1 (-0.5, 0.2) | .42 |
|  | 16wk | -0.7 (-1.1, -0.3) | **<.001** | -0.3 (-0.7, 0.1) | .12 | -0.3 (-0.6, -0.02) | **.04** |
| BASMI | 8wk | -0.5 (-0.8, -0.2) | **.001** | -0.04 (-0.3, 0.3) | >.99 | -0.5 (-0.8, -0.2) | **.005** |
|  | 16wk | -0.5 (-0.8, -0.2) | **.002** | 0.2 (-0.2, 0.5) | .58 | -0.7 (-1.1, -0.4) | **<.001** |
| ASASHI | 8wk | -1.2 (-2.0, -0.3) | **.006** | -0.4 (-1.2, 0.5) | .91 | -0.9 (-1.7, -0.1) | **.03** |
|  | 16wk | -1.3 (-2.2, -0.4) | **.001** | -0.8 (-1.7, 0.01) | .06 | -0.6 (-1.4, 0.2) | .15 |
| SF-36 PF | 8wk | 4.2 (-0.03, 8.5) | .052 | 2.0 (-2.1, 6.1) | .72 | 3.0 (0.04, 5.9) | **.047** |
|  | 16wk | 5.4 (1.6, 9.1) | **.003** | 2.3 (-1.3, 5.9) | .36 | 3.7 (1.2, 6.2) | **.004** |
| SF-36 RP | 8wk | 2.9 (-19.5, 25.2) | >.99 | 7.1 (-14.4, 28.7) | >.99 | 1.5 (-19.5, 22.5) | .89 |
|  | 16wk | 17.3 (-1.5, 36.1) | .08 | 14.3 (-3.8, 32.4) | .17 | 7.6 (-10.5, 25.7) | .41 |
| SF-36 BP | 8wk | 8.3 (2.4, 14.2) | **.003** | 4.0 (-1.7, 9.7) | .26 | 6.4 (0.8, 12.0) | **.03** |
|  | 16wk | 8.5 (3.0, 14.1) | **.001** | 6.5 (1.2, 11.9) | **.01** | 4.0 (-1.4, 9.3) | .15 |
| SF-36 GH | 8wk | 0.6 (-6.0, 7.2) | >.99 | 2.9 (-3.5, 9.2) | .82 | 3.1 (-3.6, 9.7) | .36 |
|  | 16wk | 6.7 (-0.9, 14.4) | .10 | 3.0 (-4.4, 10.4) | .94 | 10.2 (2.6, 17.7) | **.009** |
| SF-36 VT | 8wk | 2.7 (-4.1, 9.5) | >.99 | 4.6 (-1.9, 11.2) | .26 | 0.3 (-6.4, 7.0) | .92 |
|  | 16wk | 10.8 (2.4, 19.1) | **.007** | 6.3 (-1.8, 14.3) | .18 | 8.0 (0.8, 15.2) | **.03** |
| SF-36 SF | 8wk | -1.9 (-10.8, 6.9) | >.99 | -3.1 (-11.6, 5.4) | >.99 | 2.9 (-4.7, 10.6) | .45 |
|  | 16wk | 1.9 (-8.7, 12.5) | >.99 | -2.2 (-12.5, 8.0) | >.99 | 6.4 (-2.3, 15.1) | .14 |
| SF-36 RE | 8wk | 9.0 (-11.0, 28.9) | .81 | 0 (-19.2, 19.2) | >.99 | 2.1 (-17.4, 21.5) | .83 |
|  | 16wk | 25.6 (3.1, 48.1) | **.02** | 6.0 (-15.7, 27.6) | >.99 | 11.8 (-10.0, 33.6) | .28 |
| SF-36 MH | 8wk | 5.5 (-0.2, 11.3) | .06 | 3.6 (-1.9, 9.1) | .35 | 1.7 (-4.2, 7.7) | .56 |
|  | 16wk | 6.5 (-0.5, 13.4) | .08 | 4.7 (-2.0, 11.4) | .26 | 1.4 (-5.1, 7.8) | .68 |

a Mean change from baseline between the baseline and 8 or 16-wk follow-up are compared using repeated-measures analysis of variance.

b Mean between-group differences are adjusted for baseline scores of outcome variables using analysis of covariance.

Data with significant differences (*P*<.05) are presented in bold font.

ASDAS: Ankylosing Spondylitis Disease Activity Score, PGA: Patient’s global assessment, PhGA: Physician’s global assessment, BASDAI: Bath Ankylosing Spondylitis Disease Activity Index, BASFI: Bath Ankylosing Spondylitis Functional Index, BASMI: Bath Ankylosing Spondylitis Metrology Index, ASASHI: assessment of Spondyloarthritis International Society Health Index, SF36: Medical Outcome Study Short Form-36, PF: physical function, RP: Role-Physical, BP: Bodily Pain, GH: General Health, VT: Vitality, SF: Social Functioning, RE: Role-Emotional, MH: Mental Health.
