# Supplementary material for: Adherence, Efficacy, and Safety of Wearable Technology–Assisted Combined Home-Based Exercise in Chinese Patients With Ankylosing Spondylitis: Randomized Pilot Controlled Clinical Trial
Source: J Med Internet Res. 2022 Jan 18;24(1):e29703. doi: 10.2196/29703 (PMC8808346; doi:10.2196/29703)
Supplement: Multimedia Appendix 4 [file jmir_v24i1e29703_app4.docx]

**Multimedia Appendix 4.** Seventeen patient-rated items of ASAS HI in the trial phase.

|  |  | **Intervention group, n=26** | **Control group, n=28** | ***P* Value** |
| --- | --- | --- | --- | --- |
| ASAS HI-Pain | Baseline | 14 (53.8) | 18 (64.3) | 0.435 |
|  | 8wk | 7 (26.9) | 11 (39.3) | 0.336 |
|  | 16wk | 9 (34.6) | 8 (28.6) | 0.633 |
| ASAS HI-Maintaining a body position | Baseline | 4 (15.4) | 5 (17.9) | 0.808 |
|  | 8wk | 2 (7.7) | 5 (17.9) | 0.267 |
|  | 16wk | 2 (7.7) | 5 (17.9) | 0.267 |
| ASAS HI-Moving around | Baseline | 5 (19.2) | 9 (32.1) | 0.279 |
|  | 8wk | 2 (7.7) | 11 (39.3) | **0.007** |
|  | 16wk | 2 (7.7) | 7 (25.0) | 0.088 |
| ASAS HI-Toileting | Baseline | 0 | 2 (7.1) | 0.504 |
|  | 8wk | 0 | 0 | NA |
|  | 16wk | 0 | 0 | NA |
| ASAS HI-Energy and drive | Baseline | 7 (26.9) | 10 (35.7) | 0.487 |
|  | 8wk | 4 (15.4) | 9 (32.1) | 0.150 |
|  | 16wk | 3 (11.5) | 9 (32.1) | 0.069 |
| ASAS HI-Motivation | Baseline | 11 (42.3) | 14 (50.0) | 0.571 |
|  | 8wk | 7 (26.9) | 15 (53.6) | **0.046** |
|  | 16wk | 5 (19.2) | 13 (46.4) | **0.034** |
| ASAS HI-Sexual functions | Baseline | 6 (23.1) | 4 (14.3) | 0.406 |
|  | 8wk | 3 (11.5) | 3 (10.7) | >0.999 |
|  | 16wk | 3 (11.5) | 2 (7.1) | 0.931 |
| ASAS HI-Driving | Baseline | 1 (3.8) | 0 | 0.481 |
|  | 8wk | 1 (3.8) | 0 | 0.481 |
|  | 16wk | 0 | 0 | NA |
| ASAS HI-Community life | Baseline | 3 (11.5) | 3 (10.7) | >0.999 |
|  | 8wk | 1 (3.8) | 3 (10.7) | 0.612 |
|  | 16wk | 1 (3.8) | 2 (7.1) | >0.999 |
| ASAS HI-Moving around | Baseline | 0 | 0 | NA |
|  | 8wk | 1 (3.8) | 0 | 0.481 |
|  | 16wk | 0 | 1 (3.6) | >0.999 |
| ASAS HI-Handling stress | Baseline | 6 (23.1) | 7 (25.0) | 0.869 |
|  | 8wk | 5 (19.2) | 6 (21.4) | 0.841 |
|  | 16wk | 5 (19.2) | 6 (21.4) | 0.841 |
| ASAS HI-Recreation and leisure | Baseline | 3 (11.5) | 3 (10.7) | >0.999 |
|  | 8wk | 2 (7.7) | 2 (7.1) | >0.999 |
|  | 16wk | 1 (3.8) | 1 (3.6) | >0.999 |
| ASAS HI-Emotional functions | Baseline | 3 (11.5) | 3 (10.7) | >0.999 |
|  | 8wk | 2 (7.7) | 3 (10.7) | >0.999 |
|  | 16wk | 2 (7.7) | 4 (14.3) | 0.736 |
| ASAS HI-Washing oneself | Baseline | 0 | 1 (3.6) | >0.999 |
|  | 8wk | 1 (3.8) | 2 (7.1) | >0.999 |
|  | 16wk | 0 | 1 (3.6) | >0.999 |
| ASAS HI-Economic self-sufficiency | Baseline | 2 (7.7) | 1 (3.6) | 0.947 |
|  | 8wk | 0 | 2 (7.1) | 0.504 |
|  | 16wk | 1 (3.8) | 1 (3.6) | >0.999 |
| ASAS HI-Sleep | Baseline | 5 (19.2) | 4 (14.3) | 0.903 |
|  | 8wk | 3 (11.5) | 2 (7.1) | 0.931 |
|  | 16wk | 3 (11.5) | 1 (3.6) | 0.551 |
| ASAS HI-Handling stress | Baseline | 1 (3.8) | 0 | 0.481 |
|  | 8wk | 0 | 0 | NA |
|  | 16wk | 0 | 0 | NA |

ASAS HI: Assessment of Spondyloarthritis International Society Health Index.
