# Supplementary material for: Adherence, Efficacy, and Safety of Wearable Technology–Assisted Combined Home-Based Exercise in Chinese Patients With Ankylosing Spondylitis: Randomized Pilot Controlled Clinical Trial
Source: J Med Internet Res. 2022 Jan 18;24(1):e29703. doi: 10.2196/29703 (PMC8808346; doi:10.2196/29703)
Supplement: Multimedia Appendix 5 [file jmir_v24i1e29703_app5.docx]

Multimedia Appendix 5. Effects of exercise on VO_2_ max, body composition, and range of motion of cervical spine and hip joints.

| **Outcome** | **Visit** | **Intervention Group, n=26** | | **Control Group, n=28** | | **Mean Between-Group Difference in Change from Baselineb** | ***P* Valueb** |
| --- | --- | --- | --- | --- | --- | --- | --- |
|  |  | **Mean Change from Baseline** | ***P* Valuea** | **Mean Change from Baseline** | ***P* Valuea** |  |  |
| VO2max, ml/kg/min | 8wk | 0.9 (-2.2, 4.0) | >.99 | -2.1 (-5.1, 0.8) | .24 | 3.7 (0.4, 7.1) | **.03** |
|  | 16wk | -0.7 (-3.6, 2.2) | >.99 | -2.2 (-5.0, 0.6) | .17 | 2.7 (0.02. 5.3) | **.048** |
| Total lean mass, Kg | 8wk | 0.4 (0.002, 0.8) | **.048** | 0.1 (-0.3, 0.5) | >.99 | -0.3 (-0.7, 0.2) | .20 |
|  | 16wk | 0.3 (-0.1, 0.6) | .25 | 0.4 (-0.03, 0.7) | **.03** | 0.1 (-0.3, 0.5) | .58 |
| BMI, Kg/m2 | 8wk | 0.1 (-0.1, 0.3) | .32 | 0.3 (-0.1, 0.5) | **.001** | -0.2 (-0.4, 0.1) | .20 |
|  | 16wk | 0.2 (-0.1, 0.5) | .17 | 0.5 (-0.2, 0.7) | **<.001** | -0.3 (-0.6, 0.02) | .07 |
| PBF, % | 8wk | -0.1 (-1.0, 0.7) | >.99 | 0.7 (-0.1, 1.6) | .09 | -1.0 (-2.0, -0.01) | **.048** |
|  | 16wk | 0.1 (-1.0, 1.2) | >.99 | 0.7 (-0.4, 1.7) | .37 | -0.8 (-2.1, 0.5) | .21 |
| VFA, cm2 | 8wk | -0.3 (-3.3, 2.7) | >.99 | 4.5 (-0.5, 7.5) | **.001** | -4.9 (-8.5, -1.4) | **.008** |
|  | 16wk | 0.3 (-4.0, 4.7) | >.99 | 5.1 (-0.8, 9.4) | **.01** | -4.9 (-10.0, 0.3) | .06 |
| Cervical flexion, ° | 8wk | 0.4 (-5.1, 5.9) | >.99 | -0.7 (-6.0, 4.6) | >.99 | -0.5 (-6.0, 5.0) | .86 |
|  | 16wk | -2.1 (-7.0, 2.8) | .90 | -4.2 (-8.9, 0.5) | .10 | 0.9 (-4.2, 5.9) | .72 |
| Cervical extension, ° | 8wk | 7.3 (2.0, 12.6) | **.004** | 5.1 (-0.1, 10.2) | .053 | 3.3 (-2.2, 8.8) | .23 |
|  | 16wk | 7.0 (1.1, 13.0) | **.02** | 3.3 (-2.5, 9.0) | .50 | 4.3 (-2.4, 11.0) | .20 |
| Cervical lateral flexion, ° | 8wk | 2.8 (0.006, 5.6) | **.049** | 0.6 (-2.1, 3.3) | >.99 | 2.0 (-1.2, 5.2) | .20 |
|  | 16wk | 2.3 (-1.0, 5.6) | .26 | -1.9 (-5.0, 1.3) | .46 | 4.0 (0.2, 7.7) | **.04** |
| Rotation of the cervical spine, ° | 8wk | 2.3 (-0.4, 5.0) | .11 | 1.6 (-1.0, 4.2) | .39 | 1.4 (-1.1, 3.9) | .27 |
|  | 16wk | 1.2 (-2.2, 4.5) | >.99 | 2.1 (-1.1, 5.2) | .35 | -0.4 (-4.0, 3.2) | .83 |
| Hip flexion, ° | 8wk | -1.8 (-8.9, 5.2) | >.99 | -2.0 (-8.9, 4.8) | >.99 | -0.5 (-8.5, 7.4) | .90 |
|  | 16wk | -2.8 (-7.4, 1.7) | .38 | -5.3 (-9.6, -0.9) | **.01** | 1.1 (-3.0, 5.2) | .60 |
| Hip abduction, ° | 8wk | 1.5 (0.1, 2.9) | **.03** | -0.1 (-1.4, 1.3) | >.99 | 1.7 (0.2, 3.2) | **.02** |
|  | 16wk | 1.2 (-0.4, 2.8) | .20 | 0.7 (-0.8, 2.2) | .75 | 0.8 (-0.5, 2.1) | .22 |
| Hip external rotation, ° | 8wk | 0.0 (-2.0, 2.0) | >.99 | 0.0 (-1.9, 1.9) | >.99 | 0.6 (-0.8, 2.0) | .42 |
|  | 16wk | 0.0 (-2.1, 2.1) | >.99 | -0.3 (-2.3, 1.7) | >.99 | 0.9 (-0.5, 2.4) | .21 |
| Hip internal rotation, ° | 8wk | 1.3 (-1.1, 3.8) | .53 | 0.6 (-1.7, 3.0) | >.99 | 1.1 (-1.5, 3.8) | .39 |
|  | 16wk | 1.3 (-1.5, 4.0) | .79 | -1.0 (-3.7, 1.6) | >.99 | 2.7 (-0.4, 5.8) | .09 |
| Back extensor endurance test, s | 8wk | 2.9 (-13.6, 19.5) | >.99 | -0.5 (-16.5, 15.5) | >.99 | 11.3 (-6.0, 28.6) | .20 |
|  | 16wk | 9.4 (-6.0, 24.8) | .41 | -4.1 (-18.9, 10.7) | >.99 | 17.8 (0.5, 35.2) | **.04** |
| Back flexor endurance test, s | 8wk | 44.8 (7.0, 82.6) | .39 | 22.6 (-13.8, 59.0) | **.02** | 22.7 (-21.0, 66.3) | .30 |
|  | 16wk | 29.0 (-22.6, 80.5) | >.99 | -8.1 (-57.8, 41.5) | .51 | 46.7 (-10.6, 104.0) | .11 |

a Mean change from baseline between the baseline and 8 or 16-wk follow-up are compared using repeated-measures analysis of variance.

b Mean between-group differences are adjusted for baseline scores of outcome variables using analysis of covariance.

Data with significant differences (*P*<.05) are presented in bold font.

VO2max: maximal oxygen uptake; ROM: range of motion; PBF: percentage of body fat; VFA: visceral fat area.
