# Supplementary material for: Adherence, Efficacy, and Safety of Wearable Technology–Assisted Combined Home-Based Exercise in Chinese Patients With Ankylosing Spondylitis: Randomized Pilot Controlled Clinical Trial
Source: J Med Internet Res. 2022 Jan 18;24(1):e29703. doi: 10.2196/29703 (PMC8808346; doi:10.2196/29703)
Supplement: Multimedia Appendix 6 [file jmir_v24i1e29703_app6.pdf]

CONSORT-EHEALTH Checklist V1.6.2 Report	Manuscript Number	29703

(based on CONSORT-EHEALTH V1.6), available at [http://tinyurl.com/consort-ehealth-v1-6].

Date completed

06/18/2021, 1:06am

by

Yiwen Wang

The Adherence, Efficacy and Safety of Wearable Technology-Assisted Combined Hom

TITLE

1a-i) Identify the mode of delivery in the title

Wearable Technology-Assisted

1a-ii) Non-web-based components or important co-interventions in title

1a-iii) Primary condition or target group in the title

Ankylosing Spondylitis

ABSTRACT

1b-i) Key features/functionalities/components of the intervention and comparator in the METHODS section of the ABSTRACT

An exercise program consisting of moderate intensity aerobic exercise and functional exercise was given to the patients in the intervention group. The exercise
intensity was controlled by a Mio FUSE Wristband. Patients in the control group received usual care

1b-ii) Level of human involvement in the METHODS section of the ABSTRACT

We didn't add this in the abstract dut to word limit and having addresessed the
details in the method part, like "A 16-week combined exercise program consisting of
in-person counseling sessions and supervised training sessions, aerobic and functional home-based exercise was given to patients in the intervention group after randomization" and "At baseline, in-person counseling sessions were given by trained research staff"

1b-iii) Open vs. closed, web-based (self-assessment) vs. face-to-face assessments in the METHODS section of the ABSTRACT

This was a 16-week investigator-initiated, assessor-blinded, randomized, controlled trial." Morover, we have addresessed the details in the method part, like "Potential participants were identified using a passive online recruitment approach through the Smart-phone SpondyloArthritis Management System (SpAMS) which was created to provide patient education and deliver advice on disease management in patients with AS in China".

1b-iv) RESULTS section in abstract must contain use data

Seventy-seven patients were screened, of whom 55 (71%) patients were enrolled. One patient (2%) withdrew without treatment after randomization" and "The median compliance rate of the prescribed exercise protocol was 84.2% (IQR, 48.7%-97.9%)

1b-v) CONCLUSIONS/DISCUSSION in abstract for negative trials

This study is a positive trial

INTRODUCTION

2a-i) Problem and the type of system/solution

To incorporate the wearable technology-assisted home-based exercise in health care program in AS, like “Wearable technology-assisted home-based exercise has potential to serve for the standard care in AS.”

2a-ii) Scientific background, rationale: What is known about the (type of) system

“Currently, poor adherence and lack of monitoring strategy are two barriers in
improving and maintaining the quality of exercise interventions......Therefore, we conducted this clinical trial to investigate the adherence, efficacy and safety of this wearable technology-assisted combined home-based exercise intervention in patients with AS."

Does your paper address CONSORT subitem 2b?

"to investigate the adherence, efficacy and safety of this wearable technology-assisted combined home-based exercise intervention in patients with
AS

METHODS

3a) CONSORT: Description of trial design (such as parallel, factorial) including allocation ratio

This was a 16-week, randomized, open-label, accessor-blinded, controlled clinical
trial which was conducted at the Chinese PLA General Hospital".

3b) CONSORT: Important changes to methods after trial commencement (such as eligibility criteria), with reasons

No important changes to methods after trial commencement.

3b-i) Bug fixes, Downtimes, Content Changes

No major bug occured or changes were made during the 16-week trial.

4a) CONSORT: Eligibility criteria for participants

Inclusion criteria were fulfillment of the criteria for AS (1984 Modified New York criteria)18, aged 18 to 60 years, drug treatment should be stable for the preceding 1 months, ASDAS between 1.3 and 3.5".

4a-i) Computer / Internet literacy

“Exclusion criteria were......, and factors leading to the inability to receive regular exercise rehabilitation (such as language impairment, difficulty in understanding and limited movements)”

4a-ii) Open vs. closed, web-based vs. face-to-face assessments:

"Potential participants were identified using a passive online recruitment approach
through the Smart-phone SpondyloArthritis Management System...... If the
volunteers met these inclusion criteria, a face-to-face interview was conducted at the clinic to confirm all inclusion criteria finally......assessment was conducted ...... by trained research staff blinded to group assignment".

4a-iii) Information giving during recruitment

If the volunteers met these inclusion criteria, a face-to-face interview was conducted at the clinic to confirm all inclusion criteria finally" and "Informed consent was obtained from all eligible participants”.

4b) CONSORT: Settings and locations where the data were collected

This was a 16-week, randomized, open-label, accessor-blinded, controlled clinical
trial which was conducted at the Chinese PLA General Hospital".

4b-i) Report if outcomes were (self-)assessed through online questionnaires

"The enrolled patients were assessed at baseline, 8 and 16 weeks by trained research staff blinded to group assignment".

4b-ii) Report how institutional affiliations are displayed

Potential participants were identified using a passive online recruitment approach through the Smart-phone SpondyloArthritis Management System (SpAMS) which was created to provide patient education and deliver advice on disease management in patients with AS in China.16 The SpAMS was linked to WeChat (Tencent, first released in 2011) which was an instant messaging social network in China and can also be leveraged for professional purposes."

5) CONSORT: Describe the interventions for each group with sufficient details to allow replication, including how and when they were actually administered

5-i) Mention names, credential, affiliations of the developers, sponsors, and owners

This was an investigator-initiated trial and the authors declare no conflicts of interest.

5-ii) Describe the history/development process

Before this trial, we discussed the priorities, experience, and preferences with some patients diagnosed ankylosing spondylitis (AS) at their regular visits respectively, however, patients were not formally involved in this trial design

5-iii) Revisions and updating

The wristband was synchronized with smartphone application (G health, V 2.7.1) via bluetooth.

5-iv) Quality assurance methods

5-v) Ensure replicability by publishing the source code, and/or providing screenshots/screen-capture video, and/or providing flowcharts of the algorithms used

5-vi) Digital preservation

The URL of the application is "http://biz.dlcaring.com:8099/app/download.html". However, this is a study app and currently only open to our participants, therefore,
the URL of the application is not provided in the manuscript. We may consider release it as conditions allow.

5-vii) Access

Patients need not to pay to participate this trial and potential participants were identified using a passive online recruitment approach through the Smart-phone SpondyloArthritis Management System (SpAMS).

5-viii) Mode of delivery, features/functionalities/components of the intervention and comparator, and the theoretical framework

It allows users to track their exercise with instant feedbacks after exercise (see figure 1).

5-ix) Describe use parameters

"The prescribed protocol was 30 min effective aerobic exercise on 5 days/week......The functional exercise consisting of the posture exercise, range of motion exercises, resistance exercise, stability exercise and stretching exercises was prescribed for 60 min on 3 days/week."

5-x) Clarify the level of human involvement

"At baseline, in-person counseling sessions were given by trained research staff......in addition, supervised training sessions including a 30-min aerobic exercise and 60-min of functional exercise were given for two consecutive days by a physiotherapist at baseline and 8 weeks to each patient assigned to the intervention group".

5-xi) Report any prompts/reminders used

No extra emails, letters, phone calls or other prompts were provided.

5-xii) Describe any co-interventions (incl. training/support)

At baseline, in-person counseling sessions were given by trained research staff. The in-person counseling session is a structured interview containing the four domains: health benefits of exercise; overview of this exercise program; bullet points to effective
and safe exercise; how to use the wearable devices in this exercise program. In addition, supervised training sessions including a 30-min aerobic exercise and 60-min of functional exercise were given for two consecutive days by a physiotherapist at baseline and 8 weeks to each patient assigned to the intervention group. "

6a) CONSORT: Completely defined pre-specified primary and secondary outcome measures, including how and when they were assessed

The primary outcome was the between-group difference for change from baseline to 16-week of Ankylosing Spondylitis Disease Activity Score (ASDAS)."and "Besides
ASDAS, Bath Ankylosing Spondylitis Disease Activity Index (BASDAI) was assessed to reflect the disease activity. Moreover, patient's global assessment (PGA), physician's global assessment (PhGA), spinal pain-total pain, spinal pain-nocturnal pain, Bath Ankylosing Spondylitis Functional Index (BASFI) 22 and Bath Ankylosing Spondylitis Metrology Index (BASMI)23 were evaluated".

6a-i) Online questionnaires: describe if they were validated for online use and apply CHERRIES items to describe how the questionnaires were designed/deployed

The outcomes were all obtained offline.

6a-ii) Describe whether and how "use" (including intensity of use/dosage) was defined/measured/monitored

"During each session, the exercise intensity was monitored and controlled by a Mio FUSE HR monitor wristband which utilizes photoplethysmography (PPG) to measure heart rate."

6a-iii) Describe whether, how, and when qualitative feedback from participants was obtained

Instant feedbacks after exercise were given to the users (see figure 1).

6b) CONSORT: Any changes to trial outcomes after the trial commenced, with reasons

This was a 16-week, randomized, open-label, accessor-blinded, controlled clinical trial which was conducted at the Chinese PLA General Hospital".

7a) CONSORT: How sample size was determined

7a-i) Describe whether and how expected attrition was taken into account when calculating the sample size

The study was designed with a planned sample size of 54 patients with a 1:1 group allocation ratio. Sample size for the primary outcome, change in ASDAS from baseline to 16 weeks after randomization, was based on detecting a medium effect size of 0.25 and was performed with the G*Power 3.1 software. With a power of 95% or higher to detect differences between groups, 22 patients were calculated to be allocated to each group with a type I error rate of 5%. The loss to follow-up rate was assumed to be 20%. Therefore, the sample size of this trial was determined to be 27 patients in each group.

7b) CONSORT: When applicable, explanation of any interim analyses and stopping guidelines

The primary outcome was the between-group difference for change from baseline to 16-week of Ankylosing Spondylitis Disease Activity Score (ASDAS)."and "Besides
ASDAS, Bath Ankylosing Spondylitis Disease Activity Index (BASDAI) was assessed to reflect the disease activity. Moreover, patient's global assessment (PGA), physician's global assessment (PhGA), spinal pain-total pain, spinal pain-nocturnal pain, Bath Ankylosing Spondylitis Functional Index (BASFI) 22 and Bath Ankylosing Spondylitis Metrology Index (BASMI)23 were evaluated".

8a) CONSORT: Method used to generate the random allocation sequence

patients were randomly allocated to the intervention or control arm with 1:1 allocation ratio with a computer-generated randomization list which was performed by a research nurse unassociated with the intervention portion of the study"

8b) CONSORT: Type of randomisation; details of any restriction (such as blocking and block size)

In this study, patients were randomly allocated to the intervention or control arm with 1:1 allocation ratio by simple randomization with a computer-generated randomization list.

9) CONSORT: Mechanism used to implement the random allocation sequence (such as sequentially numbered containers), describing any steps taken to conceal the sequence until interventions were assigned

"The computer-generated randomization list was performed by a research nurse unassociated with the intervention portion of the study......The assessment staff and statisticians were masked to the group assignment".

10) CONSORT: Who generated the random allocation sequence, who enrolled participants, and who assigned participants to interventions

"The computer-generated randomization list was performed by a research nurse unassociated with the intervention portion of the study".

11a) CONSORT: Blinding - If done, who was blinded after assignment to interventions (for example, participants, care providers, those assessing outcomes) and how

11a-i) Specify who was blinded, and who wasn't

5

11a-ii) Discuss e.g., whether participants knew which intervention was the "intervention of interest" and which one was the "comparator"

5

11b) CONSORT: If relevant, description of the similarity of interventions

This item is not relevant for our study.

12a) CONSORT: Statistical methods used to compare groups for primary and secondary outcomes

"Separate analyses of covariance were used to determine mean between-group differences controlling for baseline level of outcomes". "The χ2 or Fisher exact tests were used to compare frequencies".

12a-i) Imputation techniques to deal with attrition / missing values

Last observation carried forward was used for missing observations".

12b) CONSORT: Methods for additional analyses, such as subgroup analyses and adjusted analyses

A sensitivity analysis (per-protocol analysis) of primary outcome was conducted with the analysis of covariance including only patients who finished the 16-week follow-up in two groups and who followed ≥80% of the prescribed exercise protocol in the intervention group".

RESULTS

13a) CONSORT: For each group, the numbers of participants who were randomly assigned, received intended treatment, and were analysed for the primary outcome

The primary and secondary outcomes were analyzed according to the intention-to-treat (ITT) principles by including all patients who were randomly allocated to either group underr went at least 1 efficacy assessment". In addition,"a sensitivity analysis (per-protocol analysis) of primary outcome was conducted with the analysis of covariance including only patients who finished the 16-week follow-up in two groups and who followed ≥80% of the prescribed exercise protocol in the intervention group".

13b) CONSORT: For each group, losses and exclusions after randomisation, together with reasons

The 8-week retention rates were 92.3% (24/26) for the intervention group, and 89.3% (25/28) for the control group. The 16-week retention rates were 88.5% (23/26) for the intervention group, and 85.7% (24/28) for the control group"

13b-i) Attrition diagram

Treatment assignments and withdrawal in the intention-to-treat population were presented in figure 2.

14a) CONSORT: Dates defining the periods of recruitment and follow-up

"The enrolled patients were assessed at baseline, 8 and 16 weeks".

**14a-i) Indicate if critical “secular events” fell into the study period**		
No critical secular events fell into the study period.		
**14b) CONSORT: Why the trial ended or was stopped (early)**		
Not applicable.		
**15) CONSORT: A table showing baseline demographic and clinical characteristics for each group**		
see Table 1		
**15-i) Report demographics associated with digital divide issues**		
See Table 1		
**16a) CONSORT: For each group, number of participants (denominator) included in each analysis and whether the analysis was by original assigned groups**		
**16-i) Report multiple “denominators” and provide definitions**		
The median adherence rate of the prescribed exercise protocol was 84.2% (IQR, 48.7%-97.9%) among all patients who were assignment to the intervention group. Sixteen (61.5%) patients in the intervention group followed ≥80% of the prescribed exercise protocol (≥103 of 128 sessions). Only two (7.7%) patients attended <10% of the prescribed exercise protocol (<13 of 128 sessions)"		
**16-ii) Primary analysis should be intent-to-treat**		
"The primary and secondary outcomes were analyzed according to the<br>intention-to-treat (ITT) principles by including all patients who were randomly allocated to either group and underwent at least 1 efficacy assessment		
**17a) CONSORT: For each primary and secondary outcome, results for each group, and the estimated effect size and its precision (such as 95% confidence interval)**		
see Table 2 and 3		
**17a-i) Presentation of process outcomes such as metrics of use and intensity of use**		
"Sixteen (61.5%) patients in the intervention group followed ≥80% of the prescribed of 128 sessions). Only two (7.7%) patients attended <10%<br>e protocol (<13 of 128 sessions)".		
**17b) CONSORT: For binary outcomes, presentation of both absolute and relative effect sizes is recommended**		
Not applicable as the outcomes were all continuous variables.		
**18) CONSORT: Results of any other analyses performed, including subgroup analyses and adjusted analyses, distinguishing pre-specified from exploratory**		
A sensitivity analysis (per-protocol analysis) of primary outcome was conducted with the analysis of covariance including only patients who finished the 16-week follow-up in two groups and who followed ≥80% of the prescribed exercise protocol in the intervention group”.		
**18-i) Subgroup analysis of comparing only users**		
A sensitivity analysis (per-protocol analysis) of primary outcome was conducted with the analysis of covariance including only patients who finished the 16-week follow-up in two groups and who followed ≥80% of the prescribed exercise protocol in the intervention group”.		
**19) CONSORT: All important harms or unintended effects in each group**		
"The incidences of adverse events (AEs) observed in the intervention group and<br>control group were 11.5% (3/26) and 0, respectively (P=.11). In the intervention group,<br>one patient reported ankle pain and two patients experienced hip pain during the exercises. About one week after the exercise plans were adapted with less jumping movements or other adjustments, the pain disappeared and these three patients all completed the intervention. No serious AEs occurred during the trial phase in both groups".		
**19-i) Include privacy breaches, technical problems**		
**19-ii) Include qualitative feedback from participants or observations from staff/researchers**		
Barriers to being active were presented in Figure 3. The results indicated that “lack<br>of time” is the most distinct barrier for these participants to overcome, then were<br>the “lack of energy” and “lack of willpower”.		
DISCUSSION		
**20) CONSORT: Trial limitations, addressing sources of potential bias, imprecision, multiplicity of analyses**		
**20-i) Typical limitations in ehealth trials**		
"Several limitations regarding the trial design merit caution. One limitation was that the patients were not blinded to the allocations which is a common limitation of non-pharmacological treatment. To reduce the bias, the primary outcome was evaluated Ybyoasusersasmnesntwstaeffrwho were unaware of the specific therapeutic regimen. In addition, this was a pilot study, and although the sample size was enough to detect a medium effect size of 0.25 as the effect size in this trial was 0.527, a clinical trial with larger sample size will provide more information about the efficacy and adherence in subgroups in AS".		
**21) CONSORT: Generalisability (external validity, applicability) of the trial findings**		
**21-i) Generalizability to other populations**		
**21-ii) Discuss if there were elements in the RCT that would be different in a routine application setting**		
**22) CONSORT: Interpretation consistent with results, balancing benefits and harms, and considering other relevant evidence**		
**22-i) Restate study questions and summarize the answers suggested by the data, starting with primary outcomes and process outcomes (use)**		
The wearable technology-assisted combined home-based exercise is feasible and<br>has beneficial effects on disease activity, physical function, spinal mobility, HRQoL,<br>ROM of cervical joints and back extensor endurance in AS"		
**22-ii) Highlight unanswered new questions, suggest future research**		
A clinical trial with larger sample size will provide more information about the efficacy and adherence in subgroups in AS".		
Other information		
**23) CONSORT: Registration number and name of trial registry**		
Trial registration: ChiCTR1900024244		
**24) CONSORT: Where the full trial protocol can be accessed, if available**		
http://www.chictr.org.cn/showproj.aspx?proj=55955		
**25) CONSORT: Sources of funding and other support (such as supply of drugs), role of funders**		
This was an investigator-initiated trial.		
**X26-i) Comment on ethics committee approval**		
"The study was approved by the ethics committee at the Chinese PLA General Hospital (S2019-118-01)".		
**x26-ii) Outline informed consent procedures**		
Informed consent was obtained from all eligible participants at our clinic.		
**X26-iii) Safety and security procedures**		
"At baseline, in-person counseling sessions were given by trained research staff.<br>The in-person counseling session is a structured interview containing the four domains: health benefits of exercise; overview of this exercise program; bullet points to effective and safe exercise; how to use the wearable devices in this exercise program".		
**X27-i) State the relation of the study team towards the system being evaluated**		
The authors delcare no conflicts on interest		
